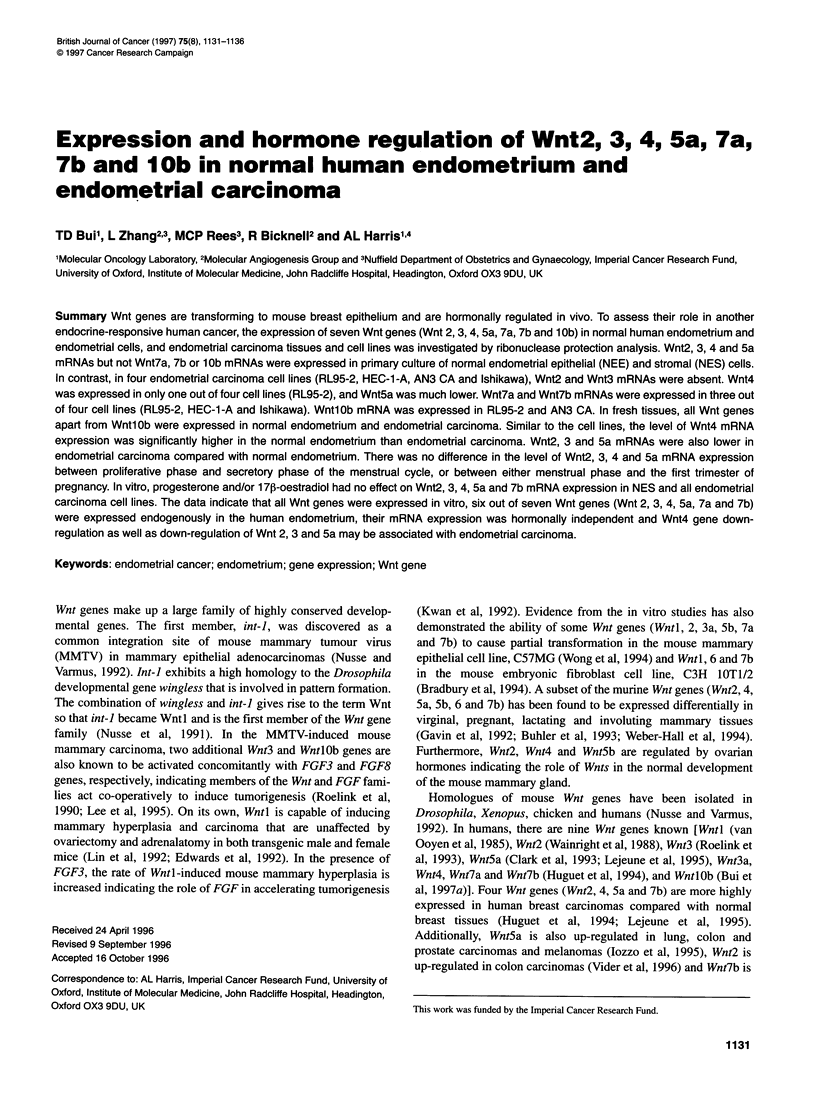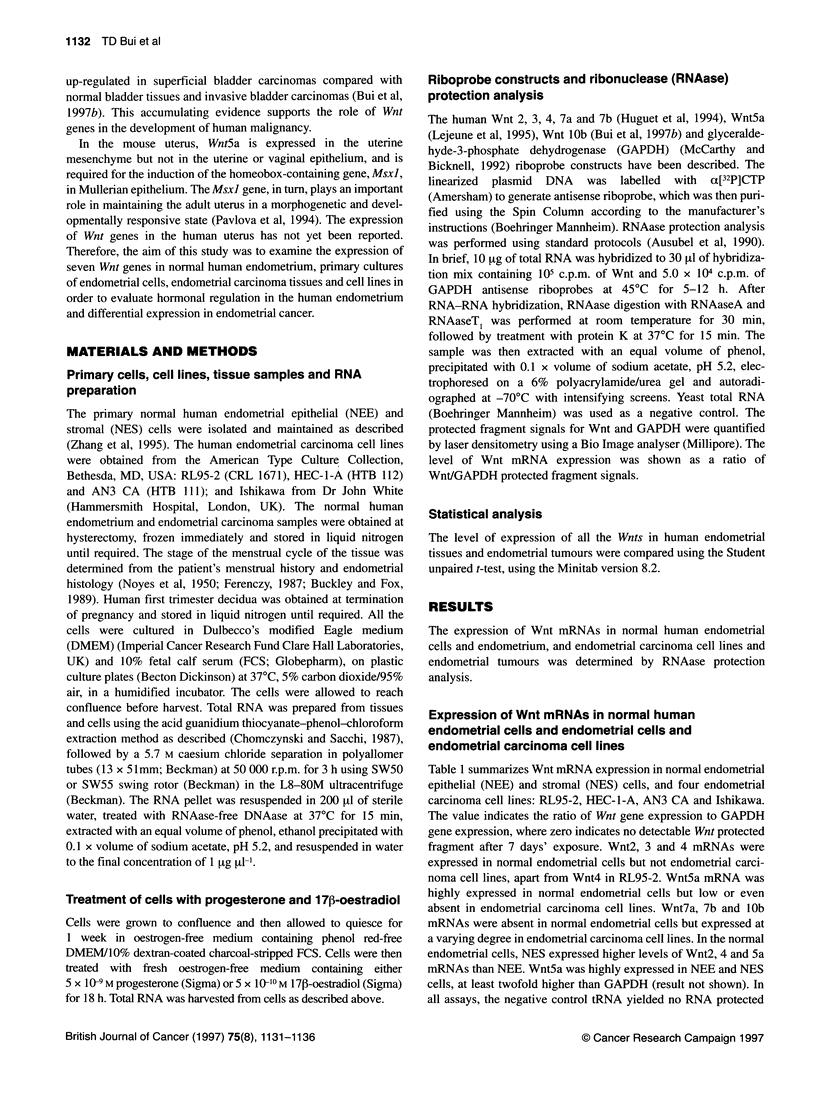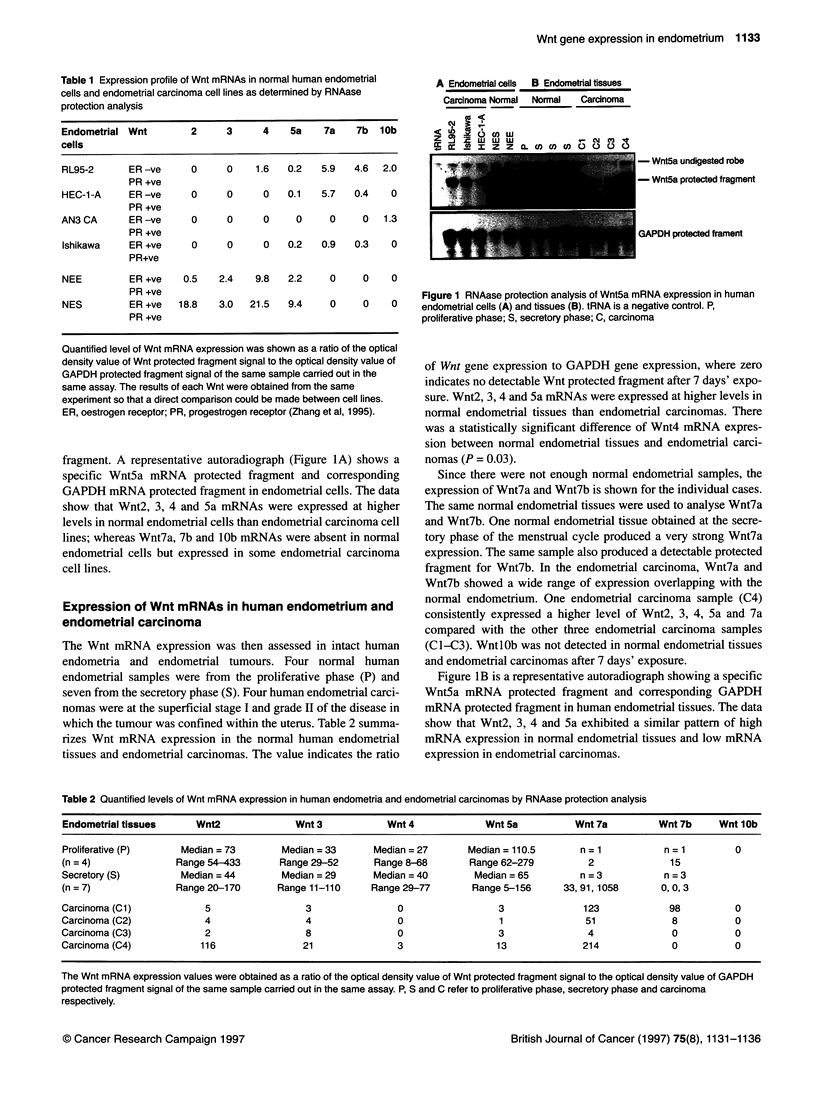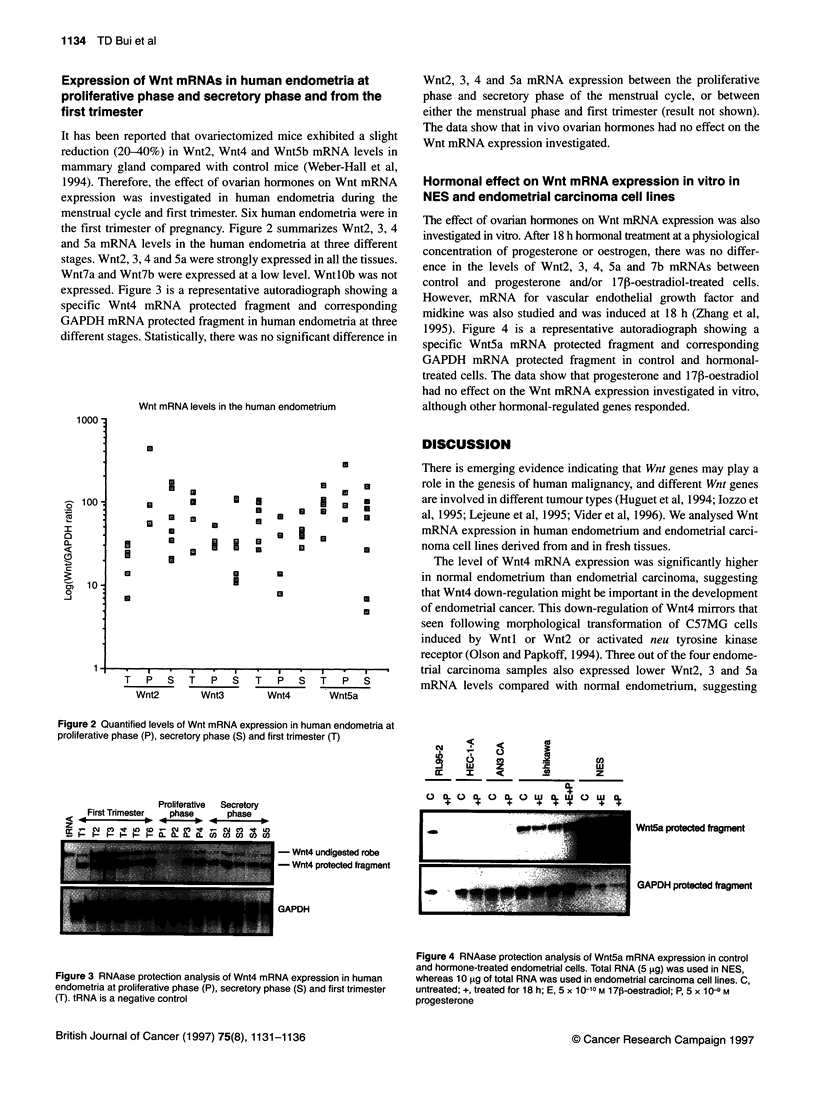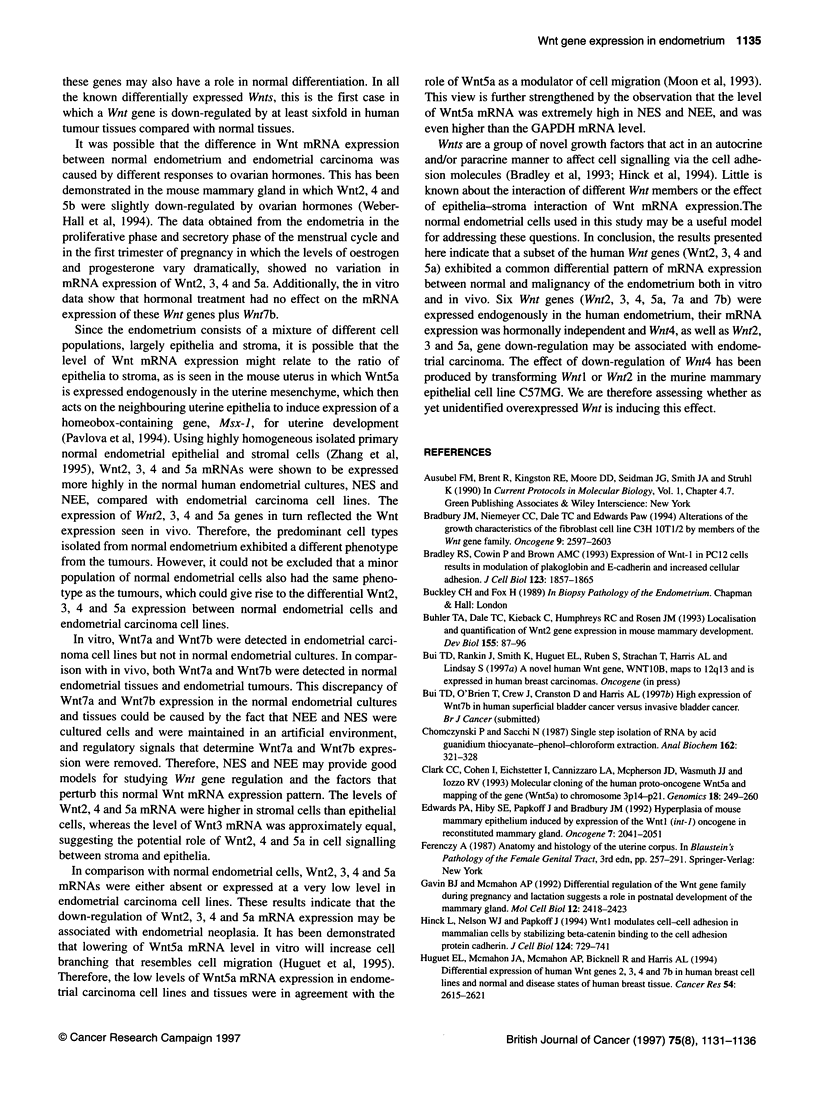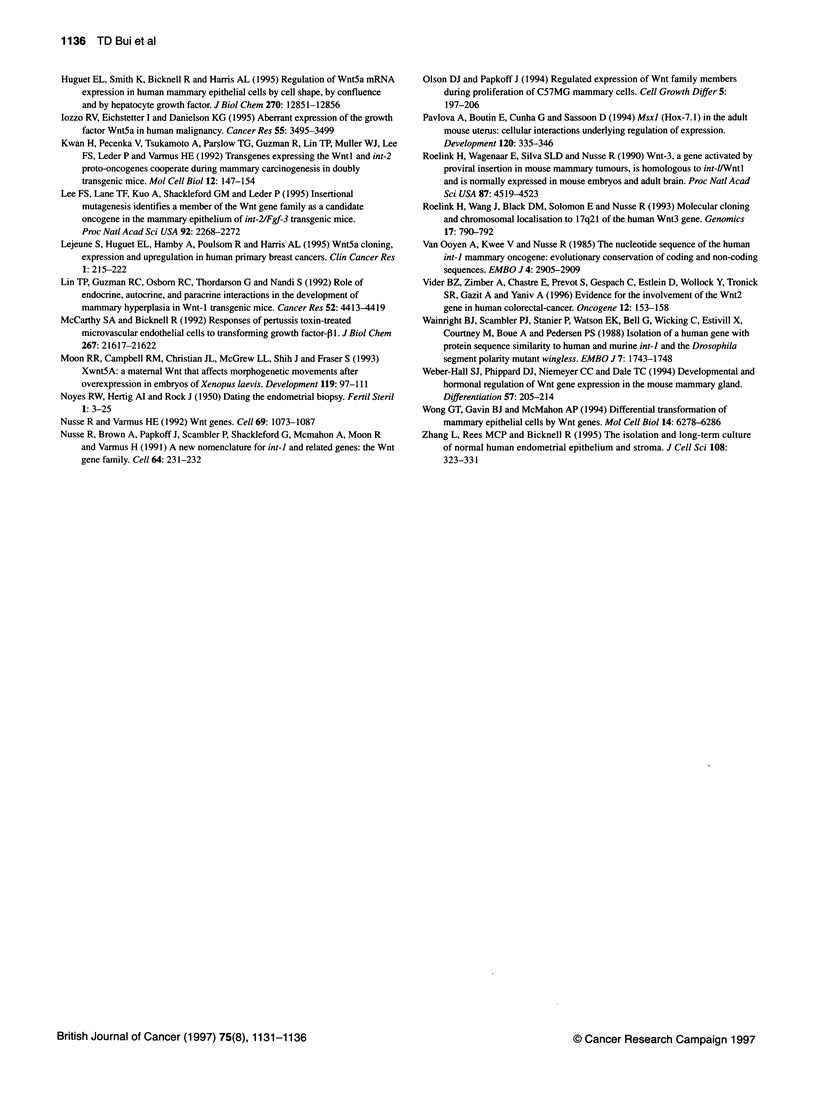# Expression and hormone regulation of Wnt2, 3, 4, 5a, 7a, 7b and 10b in normal human endometrium and endometrial carcinoma.

**DOI:** 10.1038/bjc.1997.195

**Published:** 1997

**Authors:** T. D. Bui, L. Zhang, M. C. Rees, R. Bicknell, A. L. Harris

**Affiliations:** Molecular Oncology Laboratory, University of Oxford, John Radcliffe Hospital, Headington, UK.

## Abstract

**Images:**


British Journal of Cancer (1997) 75(8), 1131–1136
© 1997 Cancer Research Campaign

### Expression and hormone regulation of Wnt2, 3, 4, 5a, 7a, 7b and 10b in normal human endometrium and endometrial carcinoma

**TD Bui[1], L Zhang[2,3], MCP Rees[3], R Bicknell[2] and AL Harris[1,4]**

[1]Molecular Oncology Laboratory, [2]Molecular Angiogenesis Group and [3]Nuffield Department of Obstetrics and Gynaecology, Imperial Cancer Research Fund, University of Oxford, Institute of Molecular Medicine, John Radcliffe Hospital, Headington, Oxford OX3 9DU, UK

**Summary** Wnt genes are transforming to mouse breast epithelium and are hormonally regulated in vivo. To assess their role in another endocrine-responsive human cancer, the expression of seven Wnt genes (Wnt 2, 3, 4, 5a, 7a, 7b and 10b) in normal human endometrium and endometrial cells, and endometrial carcinoma tissues and cell lines was investigated by ribonuclease protection analysis. Wnt2, 3, 4 and 5a mRNAs but not Wnt7a, 7b or 10b mRNAs were expressed in primary culture of normal endometrial epithelial (NEE) and stromal (NES) cells. In contrast, in four endometrial carcinoma cell lines (RL95-2, HEC-1-A, AN3 CA and Ishikawa), Wnt2 and Wnt3 mRNAs were absent. Wnt4 was expressed in only one out of four cell lines (RL95-2), and Wnt5a was much lower. Wnt7a and Wnt7b mRNAs were expressed in three out of four cell lines (RL95-2, HEC-1-A and Ishikawa). Wnt10b mRNA was expressed in RL95-2 and AN3 CA. In fresh tissues, all Wnt genes apart from Wnt10b were expressed in normal endometrium and endometrial carcinoma. Similar to the cell lines, the level of Wnt4 mRNA expression was significantly higher in the normal endometrium than endometrial carcinoma. Wnt2, 3 and 5a mRNAs were also lower in endometrial carcinoma compared with normal endometrium. There was no difference in the level of Wnt2, 3, 4 and 5a mRNA expression between proliferative phase and secretory phase of the menstrual cycle, or between either menstrual phase and the first trimester of pregnancy. In vitro, progesterone and/or 17β-oestradiol had no effect on Wnt2, 3, 4, 5a and 7b mRNA expression in NES and all endometrial carcinoma cell lines. The data indicate that all Wnt genes were expressed in vitro, six out of seven Wnt genes (Wnt 2, 3, 4, 5a, 7a and 7b) were expressed endogenously in the human endometrium, their mRNA expression was hormonally independent and Wnt4 gene down-regulation as well as down-regulation of Wnt 2, 3 and 5a may be associated with endometrial carcinoma.

**Keywords**: endometrial cancer; endometrium; gene expression; Wnt gene

*Wnt* genes make up a large family of highly conserved developmental genes. The first member, *int-1*, was discovered as a common integration site of mouse mammary tumour virus (MMTV) in mammary epithelial adenocarcinomas (Nusse and Varmus, 1992). *Int-1* exhibits a high homology to the *Drosophila* developmental gene *wingless* that is involved in pattern formation. The combination of *wingless* and *int-1* gives rise to the term Wnt so that *int-1* became Wnt1 and is the first member of the *Wnt* gene family (Nusse et al, 1991). In the MMTV-induced mouse mammary carcinoma, two additional *Wnt3* and *Wnt10b* genes are also known to be activated concomitantly with *FGF3* and *FGF8* genes, respectively, indicating members of the *Wnt* and *FGF* families act co-operatively to induce tumorigenesis (Roelink et al, 1990; Lee et al, 1995). On its own, *Wnt1* is capable of inducing mammary hyperplasia and carcinoma that are unaffected by ovariectomy and adrenalatomy in both transgenic male and female mice (Lin et al, 1992; Edwards et al, 1992). In the presence of *FGF3*, the rate of *Wnt1*-induced mouse mammary hyperplasia is increased indicating the role of *FGF* in accelerating tumorigenesis (Kwan et al, 1992). Evidence from the in vitro studies has also demonstrated the ability of some *Wnt* genes (*Wnt1*, 2, 3a, 5b, 7a and 7b) to cause partial transformation in the mouse mammary epithelial cell line, C57MG (Wong et al, 1994) and *Wnt1*, 6 and 7b in the mouse embryonic fibroblast cell line, C3H 10T1/2 (Bradbury et al, 1994). A subset of the murine *Wnt* genes (*Wnt2*, 4, 5a, 5b, 6 and 7b) has been found to be expressed differentially in virginal, pregnant, lactating and involuting mammary tissues (Gavin et al, 1992; Buhler et al, 1993; Weber-Hall et al, 1994). Furthermore, *Wnt2*, *Wnt4* and *Wnt5b* are regulated by ovarian hormones indicating the role of *Wnts* in the normal development of the mouse mammary gland.

Homologues of mouse *Wnt* genes have been isolated in *Drosophila*, *Xenopus*, chicken and humans (Nusse and Varmus, 1992). In humans, there are nine *Wnt* genes known [*Wnt1* (van Ooyen et al, 1985), *Wnt2* (Wainright et al, 1988), *Wnt3* (Roelink et al, 1993), *Wnt5a* (Clark et al, 1993; Lejeune et al, 1995), *Wnt3a*, *Wnt4*, *Wnt7a* and *Wnt7b* (Huguet et al, 1994), and *Wnt10b* (Bui et al, 1997*a*)]. Four *Wnt* genes (*Wnt2*, 4, 5a and 7b) are more highly expressed in human breast carcinomas compared with normal breast tissues (Huguet et al, 1994; Lejeune et al, 1995). Additionally, *Wnt5a* is also up-regulated in lung, colon and prostate carcinomas and melanomas (Iozzo et al, 1995), *Wnt2* is up-regulated in colon carcinomas (Vider et al, 1996) and *Wnt7b* is

Received 24 April 1996
Revised 9 September 1996
Accepted 16 October 1996

Correspondence to: AL Harris, Imperial Cancer Research Fund, University of Oxford, Institute of Molecular Medicine, John Radcliffe Hospital, Headington, Oxford OX3 9DU, UK

This work was funded by the Imperial Cancer Research Fund.

up-regulated in superficial bladder carcinomas compared with normal bladder tissues and invasive bladder carcinomas (Bui et al, 1997*b*). This accumulating evidence supports the role of *Wnt* genes in the development of human malignancy.

In the mouse uterus, *Wnt*5a is expressed in the uterine mesenchyme but not in the uterine or vaginal epithelium, and is required for the induction of the homeobox-containing gene, *Msx1*, in Mullerian epithelium. The *Msx1* gene, in turn, plays an important role in maintaining the adult uterus in a morphogenetic and developmentally responsive state (Pavlova et al, 1994). The expression of *Wnt* genes in the human uterus has not yet been reported. Therefore, the aim of this study was to examine the expression of seven *Wnt* genes in normal human endometrium, primary cultures of endometrial cells, endometrial carcinoma tissues and cell lines in order to evaluate hormonal regulation in the human endometrium and differential expression in endometrial cancer.

### MATERIALS AND METHODS

### Primary cells, cell lines, tissue samples and RNA preparation

The primary normal human endometrial epithelial (NEE) and stromal (NES) cells were isolated and maintained as described (Zhang et al, 1995). The human endometrial carcinoma cell lines were obtained from the American Type Culture Collection, Bethesda, MD, USA: RL95-2 (CRL 1671), HEC-1-A (HTB 112) and AN3 CA (HTB 111); and Ishikawa from Dr John White (Hammersmith Hospital, London, UK). The normal human endometrium and endometrial carcinoma samples were obtained at hysterectomy, frozen immediately and stored in liquid nitrogen until required. The stage of the menstrual cycle of the tissue was determined from the patient's menstrual history and endometrial histology (Noyes et al, 1950; Ferenczy, 1987; Buckley and Fox, 1989). Human first trimester decidua was obtained at termination of pregnancy and stored in liquid nitrogen until required. All the cells were cultured in Dulbecco's modified Eagle medium (DMEM) (Imperial Cancer Research Fund Clare Hall Laboratories, UK) and 10% fetal calf serum (FCS; Globepharm), on plastic culture plates (Becton Dickinson) at 37°C, 5% carbon dioxide/95% air, in a humidified incubator. The cells were allowed to reach confluence before harvest. Total RNA was prepared from tissues and cells using the acid guanidium thiocyanate–phenol–chloroform extraction method as described (Chomczynski and Sacchi, 1987), followed by a 5.7 M caesium chloride separation in polyallomer tubes (13 × 51mm; Beckman) at 50 000 r.p.m. for 3 h using SW50 or SW55 swing rotor (Beckman) in the L8–80M ultracentrifuge (Beckman). The RNA pellet was resuspended in 200 μl of sterile water, treated with RNAase-free DNAase at 37°C for 15 min, extracted with an equal volume of phenol, ethanol precipitated with 0.1 × volume of sodium acetate, pH 5.2, and resuspended in water to the final concentration of 1 $\mu g\ \mu l^{-1}$.

### Treatment of cells with progesterone and 17β-oestradiol

Cells were grown to confluence and then allowed to quiesce for 1 week in oestrogen-free medium containing phenol red-free DMEM/10% dextran-coated charcoal-stripped FCS. Cells were then treated with fresh oestrogen-free medium containing either $5 \times 10^{-9}$ M progesterone (Sigma) or $5 \times 10^{-10}$ M 17β-oestradiol (Sigma) for 18 h. Total RNA was harvested from cells as described above.

### Riboprobe constructs and ribonuclease (RNAase) protection analysis

The human Wnt 2, 3, 4, 7a and 7b (Huguet et al, 1994), Wnt5a (Lejeune et al, 1995), Wnt 10b (Bui et al, 1997*b*) and glyceraldehyde-3-phosphate dehydrogenase (GAPDH) (McCarthy and Bicknell, 1992) riboprobe constructs have been described. The linearized plasmid DNA was labelled with α[$^{32}$P]CTP (Amersham) to generate antisense riboprobe, which was then purified using the Spin Column according to the manufacturer's instructions (Boehringer Mannheim). RNAase protection analysis was performed using standard protocols (Ausubel et al, 1990). In brief, 10 μg of total RNA was hybridized to 30 μl of hybridization mix containing $10^5$ c.p.m. of Wnt and $5.0 \times 10^4$ c.p.m. of GAPDH antisense riboprobes at 45°C for 5–12 h. After RNA–RNA hybridization, RNAase digestion with RNAaseA and RNAase$T_1$ was performed at room temperature for 30 min, followed by treatment with protein K at 37°C for 15 min. The sample was then extracted with an equal volume of phenol, precipitated with 0.1 × volume of sodium acetate, pH 5.2, electrophoresed on a 6% polyacrylamide/urea gel and autoradiographed at –70°C with intensifying screens. Yeast total RNA (Boehringer Mannheim) was used as a negative control. The protected fragment signals for Wnt and GAPDH were quantified by laser densitometry using a Bio Image analyser (Millipore). The level of Wnt mRNA expression was shown as a ratio of Wnt/GAPDH protected fragment signals.

### Statistical analysis

The level of expression of all the *Wnts* in human endometrial tissues and endometrial tumours were compared using the Student unpaired *t*-test, using the Minitab version 8.2.

### RESULTS

The expression of Wnt mRNAs in normal human endometrial cells and endometrium, and endometrial carcinoma cell lines and endometrial tumours was determined by RNAase protection analysis.

### Expression of Wnt mRNAs in normal human endometrial cells and endometrial cells and endometrial carcinoma cell lines

Table 1 summarizes Wnt mRNA expression in normal endometrial epithelial (NEE) and stromal (NES) cells, and four endometrial carcinoma cell lines: RL95-2, HEC-1-A, AN3 CA and Ishikawa. The value indicates the ratio of *Wnt* gene expression to GAPDH gene expression, where zero indicates no detectable *Wnt* protected fragment after 7 days' exposure. Wnt2, 3 and 4 mRNAs were expressed in normal endometrial cells but not endometrial carcinoma cell lines, apart from Wnt4 in RL95-2. Wnt5a mRNA was highly expressed in normal endometrial cells but low or even absent in endometrial carcinoma cell lines. Wnt7a, 7b and 10b mRNAs were absent in normal endometrial cells but expressed at a varying degree in endometrial carcinoma cell lines. In the normal endometrial cells, NES expressed higher levels of Wnt2, 4 and 5a mRNAs than NEE. Wnt5a was highly expressed in NEE and NES cells, at least twofold higher than GAPDH (result not shown). In all assays, the negative control tRNA yielded no RNA protected

© Cancer Research Campaign 1997

**Table 1** Expression profile of Wnt mRNAs in normal human endometrial cells and endometrial carcinoma cell lines as determined by RNAase protection analysis

| Endometrial cells | Wnt | 2 | 3 | 4 | 5a | 7a | 7b | 10b |
|---|---|---|---|---|---|---|---|---|
| RL95-2 | ER –ve PR +ve | 0 | 0 | 1.6 | 0.2 | 5.9 | 4.6 | 2.0 |
| HEC-1-A | ER –ve PR +ve | 0 | 0 | 0 | 0.1 | 5.7 | 0.4 | 0 |
| AN3 CA | ER –ve PR +ve | 0 | 0 | 0 | 0 | 0 | 0 | 1.3 |
| Ishikawa | ER +ve PR+ve | 0 | 0 | 0 | 0.2 | 0.9 | 0.3 | 0 |
| NEE | ER +ve PR +ve | 0.5 | 2.4 | 9.8 | 2.2 | 0 | 0 | 0 |
| NES | ER +ve PR +ve | 18.8 | 3.0 | 21.5 | 9.4 | 0 | 0 | 0 |

Quantified level of Wnt mRNA expression was shown as a ratio of the optical density value of Wnt protected fragment signal to the optical density value of GAPDH protected fragment signal of the same sample carried out in the same assay. The results of each Wnt were obtained from the same experiment so that a direct comparison could be made between cell lines. ER, oestrogen receptor; PR, progestrogen receptor (Zhang et al, 1995).

**Figure 1** RNAase protection analysis of Wnt5a mRNA expression in human endometrial cells (**A**) and tissues (**B**). tRNA is a negative control. P, proliferative phase; S, secretory phase; C, carcinoma fragment. A representative autoradiograph (Figure 1A) shows a specific Wnt5a mRNA protected fragment and corresponding GAPDH mRNA protected fragment in endometrial cells. The data show that Wnt2, 3, 4 and 5a mRNAs were expressed at higher levels in normal endometrial cells than endometrial carcinoma cell lines; whereas Wnt7a, 7b and 10b mRNAs were absent in normal endometrial cells but expressed in some endometrial carcinoma cell lines.

### Expression of Wnt mRNAs in human endometrium and endometrial carcinoma

The Wnt mRNA expression was then assessed in intact human endometria and endometrial tumours. Four normal human endometrial samples were from the proliferative phase (P) and seven from the secretory phase (S). Four human endometrial carcinomas were at the superficial stage I and grade II of the disease in which the tumour was confined within the uterus. Table 2 summarizes Wnt mRNA expression in the normal human endometrial tissues and endometrial carcinomas. The value indicates the ratio of *Wnt* gene expression to GAPDH gene expression, where zero indicates no detectable Wnt protected fragment after 7 days' exposure. Wnt2, 3, 4 and 5a mRNAs were expressed at higher levels in normal endometrial tissues than endometrial carcinomas. There was a statistically significant difference of Wnt4 mRNA expression between normal endometrial tissues and endometrial carcinomas ($P = 0.03$).

Since there were not enough normal endometrial samples, the expression of Wnt7a and Wnt7b is shown for the individual cases. The same normal endometrial tissues were used to analyse Wnt7a and Wnt7b. One normal endometrial tissue obtained at the secretory phase of the menstrual cycle produced a very strong Wnt7a expression. The same sample also produced a detectable protected fragment for Wnt7b. In the endometrial carcinoma, Wnt7a and Wnt7b showed a wide range of expression overlapping with the normal endometrium. One endometrial carcinoma sample (C4) consistently expressed a higher level of Wnt2, 3, 4, 5a and 7a compared with the other three endometrial carcinoma samples (C1–C3). Wnt10b was not detected in normal endometrial tissues and endometrial carcinomas after 7 days' exposure.

Figure 1B is a representative autoradiograph showing a specific Wnt5a mRNA protected fragment and corresponding GAPDH mRNA protected fragment in human endometrial tissues. The data show that Wnt2, 3, 4 and 5a exhibited a similar pattern of high mRNA expression in normal endometrial tissues and low mRNA expression in endometrial carcinomas.

**Table 2** Quantified levels of Wnt mRNA expression in human endometria and endometrial carcinomas by RNAase protection analysis

| Endometrial tissues | Wnt2 | Wnt 3 | Wnt 4 | Wnt 5a | Wnt 7a | Wnt 7b | Wnt 10b |
|---|---|---|---|---|---|---|---|
| Proliferative (P) | Median = 73 | Median = 33 | Median = 27 | Median = 110.5 | n = 1 | n = 1 | 0 |
| (n = 4) | Range 54–433 | Range 29–52 | Range 8–68 | Range 62–279 | 2 | 15 | |
| Secretory (S) | Median = 44 | Median = 29 | Median = 40 | Median = 65 | n = 3 | n = 3 | |
| (n = 7) | Range 20–170 | Range 11–110 | Range 29–77 | Range 5–156 | 33, 91, 1058 | 0, 0, 3 | |
| Carcinoma (C1) | 5 | 3 | 0 | 3 | 123 | 98 | 0 |
| Carcinoma (C2) | 4 | 4 | 0 | 1 | 51 | 8 | 0 |
| Carcinoma (C3) | 2 | 8 | 0 | 3 | 4 | 0 | 0 |
| Carcinoma (C4) | 116 | 21 | 3 | 13 | 214 | 0 | 0 |

The Wnt mRNA expression values were obtained as a ratio of the optical density value of Wnt protected fragment signal to the optical density value of GAPDH protected fragment signal of the same sample carried out in the same assay. P, S and C refer to proliferative phase, secretory phase and carcinoma respectively.

© Cancer Research Campaign 1997 

### Expression of Wnt mRNAs in human endometria at proliferative phase and secretory phase and from the first trimester

It has been reported that ovariectomized mice exhibited a slight reduction (20–40%) in Wnt2, Wnt4 and Wnt5b mRNA levels in mammary gland compared with control mice (Weber-Hall et al, 1994). Therefore, the effect of ovarian hormones on Wnt mRNA expression was investigated in human endometria during the menstrual cycle and first trimester. Six human endometria were in the first trimester of pregnancy. Figure 2 summarizes Wnt2, 3, 4 and 5a mRNA levels in the human endometria at three different stages. Wnt2, 3, 4 and 5a were strongly expressed in all the tissues. Wnt7a and Wnt7b were expressed at a low level. Wnt10b was not expressed. Figure 3 is a representative autoradiograph showing a specific Wnt4 mRNA protected fragment and corresponding GAPDH mRNA protected fragment in human endometria at three different stages. Statistically, there was no significant difference in Wnt2, 3, 4 and 5a mRNA expression between the proliferative phase and secretory phase of the menstrual cycle, or between either the menstrual phase and first trimester (result not shown). The data show that in vivo ovarian hormones had no effect on the Wnt mRNA expression investigated.

**Figure 2** Quantified levels of Wnt mRNA expression in human endometria at proliferative phase (P), secretory phase (S) and first trimester (T)

**Figure 3** RNAase protection analysis of Wnt4 mRNA expression in human endometria at proliferative phase (P), secretory phase (S) and first trimester (T). tRNA is a negative control

### Hormonal effect on Wnt mRNA expression in vitro in NES and endometrial carcinoma cell lines

The effect of ovarian hormones on Wnt mRNA expression was also investigated in vitro. After 18 h hormonal treatment at a physiological concentration of progesterone or oestrogen, there was no difference in the levels of Wnt2, 3, 4, 5a and 7b mRNAs between control and progesterone and/or 17β-oestradiol-treated cells. However, mRNA for vascular endothelial growth factor and midkine was also studied and was induced at 18 h (Zhang et al, 1995). Figure 4 is a representative autoradiograph showing a specific Wnt5a mRNA protected fragment and corresponding GAPDH mRNA protected fragment in control and hormonal-treated cells. The data show that progesterone and 17β-oestradiol had no effect on the Wnt mRNA expression investigated in vitro, although other hormonal-regulated genes responded.

### DISCUSSION

There is emerging evidence indicating that *Wnt* genes may play a role in the genesis of human malignancy, and different *Wnt* genes are involved in different tumour types (Huguet et al, 1994; Iozzo et al, 1995; Lejeune et al, 1995; Vider et al, 1996). We analysed Wnt mRNA expression in human endometrium and endometrial carcinoma cell lines derived from and in fresh tissues.

The level of Wnt4 mRNA expression was significantly higher in normal endometrium than endometrial carcinoma, suggesting that Wnt4 down-regulation might be important in the development of endometrial cancer. This down-regulation of Wnt4 mirrors that seen following morphological transformation of C57MG cells induced by Wnt1 or Wnt2 or activated *neu* tyrosine kinase receptor (Olson and Papkoff, 1994). Three out of the four endometrial carcinoma samples also expressed lower Wnt2, 3 and 5a mRNA levels compared with normal endometrium, suggesting

**Figure 4** RNAase protection analysis of Wnt5a mRNA expression in control and hormone-treated endometrial cells. Total RNA (5 μg) was used in NES, whereas 10 μg of total RNA was used in endometrial carcinoma cell lines. C, untreated; +, treated for 18 h; E, 5 × $10^{-10}$ M 17β-oestradiol; P, 5 × $10^{-9}$ M progesterone

© Cancer Research Campaign 1997

these genes may also have a role in normal differentiation. In all the known differentially expressed *Wnts*, this is the first case in which a *Wnt* gene is down-regulated by at least sixfold in human tumour tissues compared with normal tissues.

It was possible that the difference in Wnt mRNA expression between normal endometrium and endometrial carcinoma was caused by different responses to ovarian hormones. This has been demonstrated in the mouse mammary gland in which Wnt2, 4 and 5b were slightly down-regulated by ovarian hormones (Weber-Hall et al, 1994). The data obtained from the endometria in the proliferative phase and secretory phase of the menstrual cycle and in the first trimester of pregnancy in which the levels of oestrogen and progesterone vary dramatically, showed no variation in mRNA expression of Wnt2, 3, 4 and 5a. Additionally, the in vitro data show that hormonal treatment had no effect on the mRNA expression of these *Wnt* genes plus *Wnt*7b.

Since the endometrium consists of a mixture of different cell populations, largely epithelia and stroma, it is possible that the level of Wnt mRNA expression might relate to the ratio of epithelia to stroma, as is seen in the mouse uterus in which Wnt5a is expressed endogenously in the uterine mesenchyme, which then acts on the neighbouring uterine epithelia to induce expression of a homeobox-containing gene, *Msx-1*, for uterine development (Pavlova et al, 1994). Using highly homogeneous isolated primary normal endometrial epithelial and stromal cells (Zhang et al, 1995), Wnt2, 3, 4 and 5a mRNAs were shown to be expressed more highly in the normal human endometrial cultures, NES and NEE, compared with endometrial carcinoma cell lines. The expression of *Wnt*2, 3, 4 and 5a genes in turn reflected the Wnt expression seen in vivo. Therefore, the predominant cell types isolated from normal endometrium exhibited a different phenotype from the tumours. However, it could not be excluded that a minor population of normal endometrial cells also had the same phenotype as the tumours, which could give rise to the differential Wnt2, 3, 4 and 5a expression between normal endometrial cells and endometrial carcinoma cell lines.

In vitro, Wnt7a and Wnt7b were detected in endometrial carcinoma cell lines but not in normal endometrial cultures. In comparison with in vivo, both Wnt7a and Wnt7b were detected in normal endometrial tissues and endometrial tumours. This discrepancy of Wnt7a and Wnt7b expression in the normal endometrial cultures and tissues could be caused by the fact that NEE and NES were cultured cells and were maintained in an artificial environment, and regulatory signals that determine Wnt7a and Wnt7b expression were removed. Therefore, NES and NEE may provide good models for studying *Wnt* gene regulation and the factors that perturb this normal Wnt mRNA expression pattern. The levels of Wnt2, 4 and 5a mRNA were higher in stromal cells than epithelial cells, whereas the level of Wnt3 mRNA was approximately equal, suggesting the potential role of Wnt2, 4 and 5a in cell signalling between stroma and epithelia.

In comparison with normal endometrial cells, Wnt2, 3, 4 and 5a mRNAs were either absent or expressed at a very low level in endometrial carcinoma cell lines. These results indicate that the down-regulation of Wnt2, 3, 4 and 5a mRNA expression may be associated with endometrial neoplasia. It has been demonstrated that lowering of Wnt5a mRNA level in vitro will increase cell branching that resembles cell migration (Huguet et al, 1995). Therefore, the low levels of Wnt5a mRNA expression in endometrial carcinoma cell lines and tissues were in agreement with the role of Wnt5a as a modulator of cell migration (Moon et al, 1993). This view is further strengthened by the observation that the level of Wnt5a mRNA was extremely high in NES and NEE, and was even higher than the GAPDH mRNA level.

*Wnts* are a group of novel growth factors that act in an autocrine and/or paracrine manner to affect cell signalling via the cell adhesion molecules (Bradley et al, 1993; Hinck et al, 1994). Little is known about the interaction of different *Wnt* members or the effect of epithelia–stroma interaction of Wnt mRNA expression.The normal endometrial cells used in this study may be a useful model for addressing these questions. In conclusion, the results presented here indicate that a subset of the human *Wnt* genes (Wnt2, 3, 4 and 5a) exhibited a common differential pattern of mRNA expression between normal and malignancy of the endometrium both in vitro and in vivo. Six *Wnt* genes (*Wnt*2, 3, 4, 5a, 7a and 7b) were expressed endogenously in the human endometrium, their mRNA expression was hormonally independent and *Wnt*4, as well as *Wnt*2, 3 and 5a, gene down-regulation may be associated with endometrial carcinoma. The effect of down-regulation of *Wnt*4 has been produced by transforming *Wnt*1 or *Wnt*2 in the murine mammary epithelial cell line C57MG. We are therefore assessing whether as yet unidentified overexpressed *Wnt* is inducing this effect.

### REFERENCES

Ausubel FM, Brent R, Kingston RE, Moore DD, Seidman JG, Smith JA and Struhl K (1990) In *Current Protocols in Molecular Biology*, Vol. 1, Chapter 4.7. Green Publishing Associates & Wiley Interscience: New York

Bradbury JM, Niemeyer CC, Dale TC and Edwards Paw (1994) Alterations of the growth characteristics of the fibroblast cell line C3H 10T1/2 by members of the *Wnt* gene family. *Oncogene* **9**: 2597–2603

Bradley RS, Cowin P and Brown AMC (1993) Expression of Wnt-1 in PC12 cells results in modulation of plakoglobin and E-cadherin and increased cellular adhesion. *J Cell Biol* **123**: 1857–1865

Buckley CH and Fox H (1989) *In Biopsy Pathology of the Endometrium*. Chapman & Hall: London

Buhler TA, Dale TC, Kieback C, Humphreys RC and Rosen JM (1993) Localisation and quantification of Wnt2 gene expression in mouse mammary development. *Dev Biol* **155**: 87–96

Bui TD, Rankin J, Smith K, Huguet EL, Ruben S, Strachan T, Harris AL and Lindsay S (1997*a*) A novel human Wnt gene, WNT10B, maps to 12q13 and is expressed in human breast carcinomas. *Oncogene* (in press)

Bui TD, O'Brien T, Crew J, Cranston D and Harris AL (1997*b*) High expression of Wnt7b in human superficial bladder cancer versus invasive bladder cancer. *Br J Cancer* (submitted)

Chomczynski P and Sacchi N (1987) Single step isolation of RNA by acid guanidium thiocyanate–phenol–chloroform extraction. *Anal Biochem* **162**: 321–328

Clark CC, Cohen I, Eichstetter I, Cannizzaro LA, Mcpherson JD, Wasmuth JJ and Iozzo RV (1993) Molecular cloning of the human proto-oncogene Wnt5a and mapping of the gene (Wnt5a) to chromosome 3p14–p21. *Genomics* **18**: 249–260

Edwards PA, Hiby SE, Papkoff J and Bradbury JM (1992) Hyperplasia of mouse mammary epithelium induced by expression of the Wnt1 *(int-1)* oncogene in reconstituted mammary gland. *Oncogene* **7**: 2041–2051

Ferenczy A (1987) Anatomy and histology of the uterine corpus. In *Blaustein's Pathology of the Female Genital Tract*, 3rd edn, pp. 257–291. Springer-Verlag: New York

Gavin BJ and Mcmahon AP (1992) Differential regulation of the Wnt gene family during pregnancy and lactation suggests a role in postnatal development of the mammary gland. *Mol Cell Biol* **12**: 2418–2423

Hinck L, Nelson WJ and Papkoff J (1994) Wnt1 modulates cell–cell adhesion in mammalian cells by stabilizing beta-catenin binding to the cell adhesion protein cadherin. *J Cell Biol* **124**: 729–741

Huguet EL, Mcmahon JA, Mcmahon AP, Bicknell R and Harris AL (1994) Differential expression of human Wnt genes 2, 3, 4 and 7b in human breast cell lines and normal and disease states of human breast tissue. *Cancer Res* **54**: 2615–2621

© Cancer Research Campaign 1997

Huguet EL, Smith K, Bicknell R and Harris AL (1995) Regulation of Wnt5a mRNA expression in human mammary epithelial cells by cell shape, by confluence and by hepatocyte growth factor. *J Biol Chem* **270**: 12851–12856

Iozzo RV, Eichstetter I and Danielson KG (1995) Aberrant expression of the growth factor Wnt5a in human malignancy. *Cancer Res* **55**: 3495–3499

Kwan H, Pecenka V, Tsukamoto A, Parslow TG, Guzman R, Lin TP, Muller WJ, Lee FS, Leder P and Varmus HE (1992) Transgenes expressing the Wnt1 and *int-2* proto-oncogenes cooperate during mammary carcinogenesis in doubly transgenic mice. *Mol Cell Biol* **12**: 147–154

Lee FS, Lane TF, Kuo A, Shackleford GM and Leder P (1995) Insertional mutagenesis identifies a member of the Wnt gene family as a candidate oncogene in the mammary epithelium of *int-2/Fgf-3* transgenic mice. *Proc Natl Acad Sci USA* **92**: 2268–2272

Lejeune S, Huguet EL, Hamby A, Poulsom R and Harris AL (1995) Wnt5a cloning, expression and upregulation in human primary breast cancers. *Clin Cancer Res* **1**: 215–222

Lin TP, Guzman RC, Osborn RC, Thordarson G and Nandi S (1992) Role of endocrine, autocrine, and paracrine interactions in the development of mammary hyperplasia in Wnt-1 transgenic mice. *Cancer Res* **52**: 4413–4419

McCarthy SA and Bicknell R (1992) Responses of pertussis toxin-treated microvascular endothelial cells to transforming growth factor-β1. *J Biol Chem* **267**: 21617–21622

Moon RR, Campbell RM, Christian JL, McGrew LL, Shih J and Fraser S (1993) XwntSA: a maternal Wnt that affects morphogenetic movements after overexpression in embryos of *Xenopus laevis*. *Development* **119**: 97–111

Noyes RW, Hertig AI and Rock J (1950) Dating the endometrial biopsy. *Fertil Steril* **1**: 3–25

Nusse R and Varmus HE (1992) Wnt genes. *Cell* **69**: 1073–1087

Nusse R, Brown A, Papkoff J, Scambler P, Shackleford G, Mcmahon A, Moon R and Varmus H (1991) A new nomenclature for *int-1* and related genes: the Wnt gene family. *Cell* **64**: 231–232

Olson DJ and Papkoff J (1994) Regulated expression of Wnt family members during proliferation of C57MG mammary cells. *Cell Growth Differ* **5**: 197–206

Pavlova A, Boutin E, Cunha G and Sassoon D (1994) *Msx1* (Hox-7.1) in the adult mouse uterus: cellular interactions underlying regulation of expression. *Development* **120**: 335–346

Roelink H, Wagenaar E, Silva SLD and Nusse R (1990) Wnt-3, a gene activated by proviral insertion in mouse mammary tumours, is homologous to *int-l*/Wnt1 and is normally expressed in mouse embryos and adult brain. *Proc Natl Acad Sci USA* **87**: 4519–4523

Roelink H, Wang J, Black DM, Solomon E and Nusse R (1993) Molecular cloning and chromosomal localisation to 17q21 of the human Wnt3 gene. *Genomics* **17**: 790–792

Van Ooyen A, Kwee V and Nusse R (1985) The nucleotide sequence of the human *int-1* mammary oncogene: evolutionary conservation of coding and non-coding sequences. *EMBO J* **4**: 2905–2909

Vider BZ, Zimber A, Chastre E, Prevot S, Gespach C, Estlein D, Wollock Y, Tronick SR, Gazit A and Yaniv A (1996) Evidence for the involvement of the Wnt2 gene in human colorectal-cancer. *Oncogene* **12**: 153–158

Wainright BJ, Scambler PJ, Stanier P, Watson EK, Bell G, Wicking C, Estivill X, Courtney M, Boue A and Pedersen PS (1988) Isolation of a human gene with protein sequence similarity to human and murine *int-1* and the *Drosophila* segment polarity mutant *wingless*. *EMBO J* **7**: 1743–1748

Weber-Hall SJ, Phippard DJ, Niemeyer CC and Dale TC (1994) Developmental and hormonal regulation of Wnt gene expression in the mouse mammary gland. *Differentiation* **57**: 205–214

Wong GT, Gavin BJ and McMahon AP (1994) Differential transformation of mammary epithelial cells by Wnt genes. *Mol Cell Biol* **14**: 6278–6286

Zhang L, Rees MCP and Bicknell R (1995) The isolation and long-term culture of normal human endometrial epithelium and stroma. *J Cell Sci* **108**: 323–331

© Cancer Research Campaign 1997